# Mobile chest X-ray manifestations of 54 deceased patients with coronavirus disease 2019: Retrospective study

**DOI:** 10.1097/MD.0000000000023167

**Published:** 2020-11-13

**Authors:** Chunlin Xiang, Lu Huang, Liming Xia

**Affiliations:** Department of Radiology, Tongji Hospital, Tongji Medical College, Huazhong University of Science and Technology, Wuhan, China.

**Keywords:** COVID-19, SARS-CoV-2, death, chest X-ray, manifestation, pandemic

## Abstract

To describe the mobile chest X-ray manifestations of deceased patients with coronavirus disease 2019 (COVID-19).

In this retrospective study, we analyzed in patients with COVID-19 from Tongji Hospital (Wuhan, China), who had been died between February 18 and March 25, 2020. Two radiologists analyzed the radiologic characteristics of mobile chest X-ray, and analyzed the serial X-ray changes.

Fifty-four deceased patients with COVID-19 were included in the study. We found that 50 (93%) patients with lesions occurred in the bilateral lung, 4 (7%) patients occurred in the right lung, 54 (100%) patients were multifocal involvement. The number of lung fields involved was 42 (78%) patients in 6 fields, 3 (6%) patients in 5 lung fields, 4 (7%) patients in 4 lung fields, and 5 (9%) patients in 3 lung fields. Fifty-three (98%) patients had patchy opacities, 3 (6%) patients had round or oval solid nodules, 9 (17%) patients had fibrous stripes, 13 (24%) patients had pleural effusion, 8 (15%) patients had pleural thickening, 6 (11%) patients had pneumothorax, 3 (6%) patients had subcutaneous emphysema. Among the 24 patients who had serial mobile chest X-rays, 16 (67%) patients had the progression of the lesions, 8 (33%) patients had no significant change of the lesions, and there was no case of reduction of the lesions.

The mobile chest X-ray manifestations of deceased patients with COVID-19 were mostly bilateral lung, multifocal involvement, and extensive lung field, and pleural effusion, pleural thickening, and pneumothorax probably could be observed. The serial mobile chest X-ray showed that the chest lesions were progressive with a high probability.

## 1 Introduction

Coronavirus disease 2019 (COVID-19) is caused by severe acute respiratory syndrome coronavirus 2 (SARS-CoV-2), which mainly causes respiratory system of infectious disease.^[1]^ On March 11, 2020, the World Health Organization (WHO) officially declared that COVID-19 can be characterized as a pandemic due to the alarming levels of spread and severity.^[2]^ As of May 19, 2020, COVID-19 has caused more than 4600,000 confirmed cases and more than 310,000 deaths in more than 200 countries worldwide.^[3]^ In the face of this pandemic disaster, there is an urgent need for more clinical study of COVID-19 to be shared globally. Chest X-ray and chest CT played an important role in detection and diagnosis of COVID-19.^[4]^ At present, there are some previous studies^[5–7]^ on chest imaging of COVID-19, but these studies mainly focused on chest CT and survivors. Although it is reported that chest CT is a good choice for the detection and diagnosis of mild and moderate COVID-19,^[8,9]^ we should notice the critical case that can not take CT scanning by autonomous movement. So we try to the chest X-ray diagnosis protocol for critical COVID-19 because X-ray is easier to move. At the same time, X-ray is cheaper and more time-saving suitable for severe outbreak area. As far as we know, there was no complete data about chest imaging manifestations of deceased patients, especially the recent chest imaging manifestations before death. Facing the terrible increasing number of deaths globally, we must to urgent share the chest imaging findings of deceased patients with COVID-19.

In this study, we included inpatients with confirmed COVID-19 from Tongji Hospital (Wuhan, China) who had been died between February 18 and March 25, 2020. We included the mobile chest X-ray during intensive care to study, aiming to share the recent X-ray manifestations of COVID-19 before death.

## 2 Methods

### 2.1 Study design and participants

This retrospective study was performed on inpatients with confirmed COVID-19 from Wuhan Tongji Hospital, which is one of centralized admission hospitals assigned by the Chinese government for COVID-19. Demographic and clinical characteristic data were extracted from electronic medical records, and X-ray image data was extracted from picture archiving and communication systems (PACS). In this retrospective study, we included inpatients who died between February 18 and March 25, 2020, all of whom were diagnosed with COVID-19 by SARS-CoV-2 nucleic acid test (reverse transcription–polymerase chain reaction). A total of 60 patients were enrolled in the study. After excluding patients who had not had a mobile chest X-ray within one week before death, 54 patients were included finally. Twenty-four of the patients underwent 2 or more mobile chest X-rays before death. Our institutional ethics committee (Tongji Hospital, Wuhan, China) approved this retrospective study and waived written informed consent.

### 2.2 Mobile chest X-ray

All images were obtained on a mobile X-ray system (uDR370i, United Imaging, China) with patients in supine or semi supine position. The tube voltage is 78 kV, and the tube current is 3.2 mAs.

### 2.3 Image analysis

Two radiologists (LQ and HL, with 3 and 10 years of experience, respectively) reviewed the chest X-rays of all patients and unanimously determined the final chest X-ray manifestation at the survival of the COVID-19 patient, and analyzed the temporal changes in the lesions of the serial X-rays. The imaging manifestations observed by the 2 radiologists included the distribution of lesions (left, right / bilateral lungs), and the number of lesions (unifocal/ multifocal involvement). The lung fields were divided into 6 parts: upper left, middle left, lower left, upper right, middle right, and lower right, and the number of lung fields involved by lesions were counted. Two radiologists described the morphological characteristics of the lesions (patchy opacity, round or oval solid nodule, fibrous stripe), analyzed the occurrence of pleural effusion, pleural thickening, pneumothorax, and subcutaneous emphysema and the cases of “white lung.” Two radiologists also analyzed the serial X-ray changes (progression, no significant change, and reduction). Progression is defined as the number of opacity increased, the region of opacity enlarged, the density of opacity increased. Reduction is defined as the number of opacity decreased, the region of opacity reduced, the density of opacity decreased.

### 2.4 Statistical analysis

The categorical variables were presented as numbers and percentages, and the continuous variables were presented as median and interquartile range (IQR).

## 3 Results

### 3.1 Demographics and clinical characteristics of 54 deceased patients

As showed in Table 1, the median age of death was 67 (IQR 62-75) years old, and 43 (80%) patients were 60 years old and above. Male sex was in the majority with 39 (72%). The common symptoms of onset were fever 46 (85%), cough 39 (72%), dyspnea 25 (46%), and fatigue 24 (44%). Overall, 41 (76%) patients had at least 1 chronic medical disease. The common comorbidities were hypertension 23 (43%), diabetes 11 (20%), coronary heart disease 10 (19%), and chronic lung disease 8 (15%). The median time from onset of symptom to death was 30 (IQR 23-35) days. The median time from hospital admission to death was 17 (IQR 11-22) days. In the treatment, 22 (41%) patients received noninvasive ventilator, 41 (76%) patients received invasive ventilator and 2 (4%) patients received extracorporeal membrane oxygenation.

**Table 1:** Demographics and clinical characteristics of 54 deceased patients with COVID-19.

	Patients (n = 54)
**Median age, ys (IQR)**	67 (62-75)
≥60	43 (80%)
**Sex**	
Male	39 (72%)
Female	15 (28%)
**Symptoms of onset**	
Fever	46 (85%)
Cough	39 (72%)
Dyspnoea	25 (46%)
Fatigue	24 (44%)
Chest tightness	11 (20%)
Myalgia	6 (11%)
Vomiting	3 (6%)
Diarrhoea	10 (19%)
Headache	9 (17%)
**Comorbidities**	
Hypertension	23 (43%)
Diabetes	11 (20%)
Coronary heart disease	10 (19%)
Chronic lung diseases	8 (15%)
Malignancy	2 (4%)
Chronic renal failure	1 (2%)
Smoking history	20/40 (50%)
Male smoker	19/27 (70%)
Female smoker	1/13 (8%)
**Treatments**	
Non-invasive mechanical ventilation	22 (41%)
Invasive mechanical ventilation	41 (76%)
ECMO	2 (4%)
**Time of outcomes**	
Median (IQR) time from onset of symptom to death, days	30 (23-35)
Median (IQR) time from hospital admission to death, days	17 (11-22)
Median (IQR) time from final chest X-ray to death, days	3 (1-4.25)

### 3.2 Chest X-ray manifestations of 54 deceased patients

Fifty-four patients had a total of 106 chest X-rays. The median time from final chest X-ray to death was 3 (IQR 1-4.25) days. We found that 50 (93%) patients with lesions occurred in bilateral lung, 4 (7%) patients occurred in right lung, 54 (100%) patients were multifocal involvement. The number of lung fields involved was 42 (78%) patients in 6 fields, 3 (6%) patients in 5 lung fields, 4 (7%) in 4 lung fields, and 5 (9%) in 3 lung fields. Fifty-three (98%) patients had patchy opacities, 3 (6%) patients had round or oval solid nodules, 9 (17%) patients had fibrous stripes, 13 (24%) patients had pleural effusion, 8 (15%) patients had pleural thickening, 6 (11%) patients had pneumothorax, 3 (6%) patients had subcutaneous emphysema, 11 (20%) patients were “white lung.” Table 2 shows the details.

**Table 2:** Chest X-ray manifestations of 54 deceased patients with COVID-19.

	Patients (n = 54)
**Distribution of lesions**	
Bilateral	50 (93%)
Left	0
Right	4 (7%)
**Number of lesions**	
Unifocal involvement	0
Multifocal involvement	54 (100%)
**Number of lung fields involved**	
1	0
2	0
3	5 (9%)
4	4 (7%)
5	3 (6%)
6	42 (78%)
More than three lung fields involved	54 (100%)
Patchy opacities	53 (98%)
Round or oval solid nodules	3 (6%)
Fibrous stripes	9 (17%)
Pleural effusion	13 (24%)
Pleural thickening	8 (15%)
Pneumothorax	6 (11%)
Subcutaneous emphysema	3 (6%)
White lung	11 (20%)

### 3.3 Serial chest X-ray changes of 24 deceased patients

Twenty-four patients with undergoing serial chest X-rays had a total of 76 x–rays, the median number of X-rays was 3 (IQR 2-3.75), the median time from first chest X-ray to death was 8 (IQR 6-11.5) days, and the median time from final chest X-ray to death was 3 (IQR 1-3.75) days. Sixteen (67%) patients had the progression of the lesions, 8 (33%) patients had no significant change of the lesions, and there was no case of reduction of the lesions. Table 3 shows the details.

**Table 3:** Serial chest X-ray manifestations of 24 deceased patients with COVID-19.

	First chest X-ray (n = 24)	Final chest X-ray (n = 24)
Median (IQR) time from the chest X-ray to death, days	8 (6–11.5)	3 (1–3.75)
**Distribution of lesions**		
Bilateral	23 (96%)	23 (96%)
Left	0	0
Right	1 (4%)	1 (4%)
**Number of lesions**		
Unifocal involvement	0	0
Multifocal involvement	24 (100%)	24 (100%)
**Number of lung fields involved**		
1	0	0
2	0	0
3	2 (8%)	1 (4%)
4	4 (17%)	1 (4%)
5	5 (21%)	2 (8%)
6	13 (54%)	20 (83%)
More than three lung fields involved	24 (100%)	24 (100%)
Patchy opacities	24 (100%)	24 (100%)
Round or oval solid nodules	1 (4%)	1 (4%)
Fibrous stripes	3 (13%)	4 (17%)
Pleural effusion	3 (13%)	7 (29%)
Pleural thickening	2 (8%)	2 (8%)
Pneumothorax	0	3 (13%)
Subcutaneous emphysema	0	2 (8%)
White lung	0	6 (25%)
Progression of X-ray manifestations	16/24 (67%)	
No significant change of X-ray manifestations	8/24 (33%)	
Reduction of X-ray manifestations	0	

## 4 Discussion

COVID-19 mainly caused a typical lung injury from viral pneumonia. Previous chest CT studies have provided some detailed descriptions of the chest imaging of survivors, Pan et al^[10]^ found that chest imaging changes were very rapid in COVID-19 patients. It is essential to pay attention to the follow-up changes of the chest imaging, COVID-19 patients are usually critically ill during intensive care, in order to provide humanistic care for critically ill patients, mobile X-ray is used for imaging instead of patients being forced to move to X-ray room or CT room.

**Figure 1:**
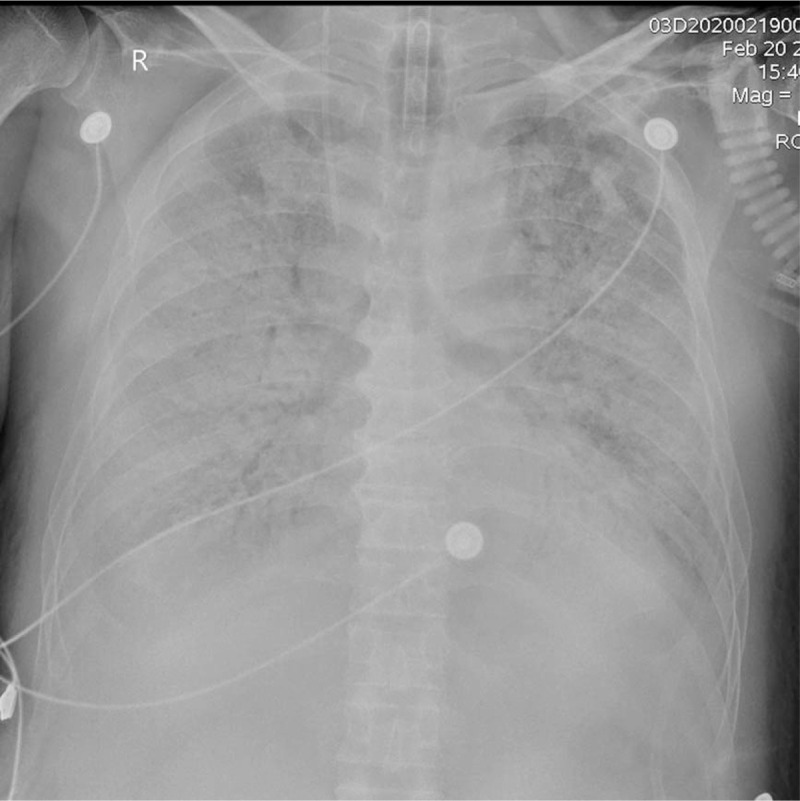
Male, 69 years old. There were multiple areas of opacification in both lungs, showing diffuse high-density patchy opacity with uneven density, presenting “white lung.” The patient died 4 hours later after the X-ray.

In our study, we found that the chest X-ray of the deceased patients were mainly bilateral distribution of the lesions, all lesions were multifocal involvement, all of patients had more than 3 lung fields involved, and 78% of the patients had 6 lung fields involved, mainly presenting as patchy opacities in morphology. Patchy opacities showed diffuse changes and consolidated with each other. The density of both lungs increased significantly, invading the whole lung field and presenting “white lung” (Fig. 1). According to the latest CT longitudinal study,^[11]^ the extent of CT abnormalities progressed rapidly after the onset of symptoms, peaked around 6 to 11 days, and followed by persistence of high levels with lung abnormalities. The median time from onset of symptoms to death was 30 days in our study, chest lesions presented multifocal involvement and extensive lung field due to long-term and sustained lung damage. In the critical stage of COVID-19, the lesions were extensively infiltrated and the alveoli were filled with exudate caused by inflammation, leading to necrotizing bronchitis and diffuse alveolar injury, and the respiratory function was weakened to failure,^[12]^ The extensive distribution of the lesions and even the appearance of “white lung” are often the indications of critical illness and poor prognosis.

Pleural effusion was found in 13 (24%) patients, which was higher than that of 5% of the patients in the latest study.^[7]^ Based on the experience of Middle East respiratory syndrome coronavirus (MERS-CoV) infection,^[13]^ the appearance of pleural effusion may predict a poor prognosis in COVID-19. Pneumothorax was found in 6 (11%) patients and subcutaneous emphysema in 3 (6%) patients, which is very rare in the early stage or mild disease of COVID-19.^[6,7]^ During the critical stage, the alveolar structure and function of the patient were severely damaged, and the blood and gas exchange in the lung was obstructed. It may be that mechanical ventilation promoted the occurrence of pneumothorax or subcutaneous emphysema. Similarly, the appearance of pneumothorax or subcutaneous emphysema is a sign of the impairment of lung function. Pneumothorax will compress the adjacent area and damage the cardiopulmonary system.^[14]^ For critically ill patients, the appearance of pneumothorax or subcutaneous emphysema predicted a poor short-term prognosis. Round or oval solid nodules were rarely found. COVID-19 was mainly multifocal involved in the chest in the critical stage of the disease, and lesions fused into a patchy distribution. Fibrous stripes were found in 9 (17%) patients. Fibrous stripes were generally considered as the manifestation of pulmonary fibrosis,^[15]^ and it is the cell components gradually replaced by scar tissue, but the relationship between fibrosis and patients’ prognosis is debatable.^[16]^ Pleural thickening was found in 8 (15%) patients, including interlobular septal thickening. In the early stage of COVID-19, the lesions were mainly located in the peripheral areas under the pleura. With the progress of the disease, the invasion of the pleura would also increase. It was found that pleural thickening was more common in severe or critical patients than in ordinary patients.^[17]^

**Figure 2:**
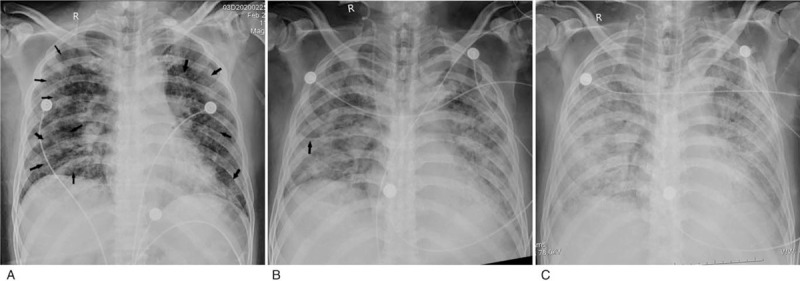
A 51-year-old male with 3 mobile chest X-rays before death. A. The first X-ray showed small fluffy patchy opacities scattered in bilateral lung fields (arrows), invading 6 lung fields. B. Seven days after the first X-ray, the second X-ray showed that the multiple areas of opacification increased, and the right horizontal fissure thickened (arrow). C. Four days after the second X-ray, the third X-ray showed that the multiple areas of opacification further increased, and patchy opacities were diffusely distributed in all lung fields, presenting “white lung.” The patient died 2 days after the third X-ray.

**Figure 3:**
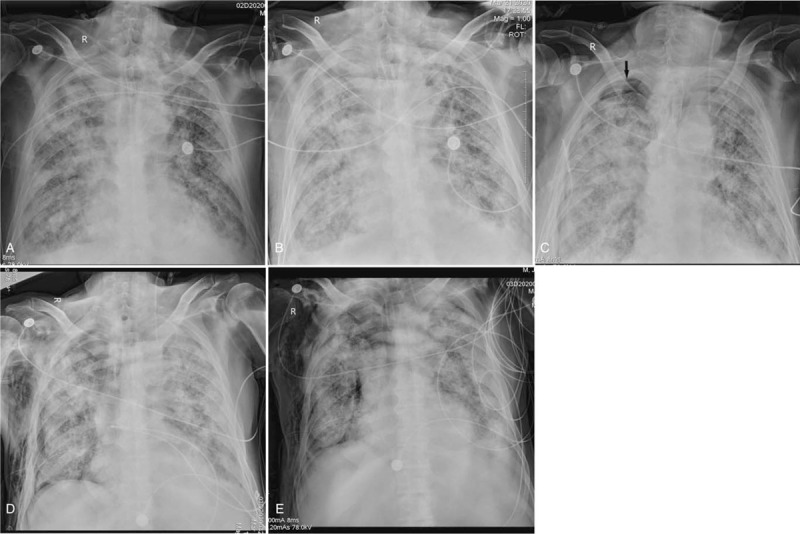
A 67-year-old male with 5 mobile chest X-rays before death. This series of X-rays showed the rapid progress of the lesions and the appearance of pneumothorax and subcutaneous emphysema. A. The first X-ray, extensive patchy opacities can be seen in both lung fields, involving the entire lung fields. B. Two days after the first X-ray, the second X-ray showed that patchy opacities increased in density and profusion. C. Three days after the second X-ray, the third X-ray showed that pneumothorax in the right upper lung field (arrow) appeared. D. Three days after the third X-ray, the fourth X-ray showed that the region of the right pneumothorax was enlarged, and the right subcutaneous emphysema appeared. E. One day after the fourth X-ray, the fifth X-ray showed that the region of the pneumothorax and subcutaneous emphysema was further enlarged. The patient died 1 day after the fifth X-ray.

Through serial chest X-ray analysis, most of the X-ray manifestations are progressive, mainly manifested in the region of opacity enlarged, the density of opacity increased, as well as the appearance of pneumothorax and subcutaneous emphysema. 6 cases of “white lung” occurred during serial X-ray follow-up. We found none of the findings in the first X-ray. Patchy opacities gradually enlarged and increased until it diffused in all lung fields, presenting “white lung.” Serial X-ray observations showed that patients died soon after the lesions progressed to “white lung” (Fig. 2). Three cases of pneumothorax and 2 cases of subcutaneous emphysema occurred during serial X-ray follow-up, we found none of these findings in the first X-ray. Serial X-ray observations also showed that patients died soon after the onset of pneumothorax or subcutaneous emphysema (Fig. 3). The progressive X-ray manifestations may predict a short-term poor prognosis. The doctors should pay close attention to the development and change of chest X-ray. 33% of patients had no significant change of the lesions. The possible explanation is that COVID-19 is involved in multiple organs of the whole body, and some patients have malignant progress other than the chest lesions. Therefore, in the face of critical patients with COVID-19, the attention to the progress of the disease is not only the chest lesions, but also the multi organs of the whole body.

In our study, the common symptoms of onset were fever, cough, dyspnea, and fatigue. We observed that the incidence of dyspnea was 46%, higher than 31% reported in the previous study,^[18]^ it is possible that in the death cases, the patient was already in a more critical condition when he was admitted to hospital. In our study, the median age of the deceased patients was 67 (IQR 62-75), 72% of whom were males. The most common comorbidities were hypertension, diabetes, coronary heart disease, and chronic lung disease, which was similar to the study of Zhou et al.^[19]^ One of the possible reasons for this phenomenon might be that the lung aging is associated with an inability of lung cells and multiple structural and functional changes in the respiratory tract, giving rise to decreased lung function, altered pulmonary remodeling, diminished regeneration, and enhanced susceptibility to pulmonary disease, having a higher risk of ARDS development.^[20]^ Interestingly, in our study, the chronic lung disease in the comorbidities is 15%, higher than 7% reported by Zhou et al, which may be related to our highly selected deceased patients with having chest X-ray, patients with chronic lung disease may be more prone to lung symptoms, and have more urgent chest X-ray needs. Our study found that 74% of the male patients who died were smokers, and that males with a history of smoking should also be concerned about the poor prognosis.

In routine clinical practice, when confronted with a very sick patient with respiratory symptoms, the role of the mobile chest X-ray is to establish the presence of chest disease and progression of chest disease immediately. In COVID-19 outbreak areas, physicians were able to have a comprehensive understanding of the chest X-ray manifestations of the deceased patients through our study. In intensive care unit (ICU), physicians need to pay more attention to the poor prognosis of patients when chest X-ray manifestations are serious or progressive. Clinical deterioration of cases admitted to the ICU is manifested by progression of pulmonary infiltrates on chest X-ray. As clinical deterioration appears to be closely mirrored by the development of progressively worsening radiographical opacity. Close monitoring of disease progress in the ICU is important to detect deterioration. Due to the use of mechanical ventilation, physicians need to pay close attention to the occurrence of pneumothorax and subcutaneous emphysema through chest X-ray. Avoiding mechanical ventilation as much as possible and if required, utilizing low-volume, low-pressure ventilation would seem prudent.

This study has some limitations. First of all, the changes of serial chest X-rays were based on the subjective visual evaluation of radiologists, and no objective quantitative evaluation method was included. In addition, due to the limitation of the image quality of mobile chest X-ray, we have not made a detailed study of some subtle imaging manifestations.

In conclusion, we studied the mobile chest X-ray manifestations of 54 deceased patients with COVID-19, most of whom were elderly males with comorbidities. The mobile chest X-ray manifestations of deceased patients with COVID-19 were mostly bilateral lung, multifocal involvement, and extensive lung field, and pleural effusion, pleural thickening, and pneumothorax probably could be observed. The serial mobile chest X-ray showed that the chest lesions were progressive with a high probability.

## Author contributions

**Conceptualization:** Chunlin Xiang, Liming Xia.

**Data curation:** Chunlin Xiang, Lu Huang.

**Formal analysis:** Chunlin Xiang, Liming Xia.

**Investigation:** Chunlin Xiang, Liming Xia.

**Methodology:** Chunlin Xiang.

**Project administration:** Chunlin Xiang.

**Resources:** Chunlin Xiang.

**Validation:** Chunlin Xiang.

**Visualization:** Chunlin Xiang.

**Writing – original draft:** Chunlin Xiang.

**Software:** Lu Huang, Liming Xia.

**Funding acquisition:** Liming Xia.

**Supervision:** Liming Xia.

**Writing – review & editing:** Liming Xia.
